# Making the leap from structure to mechanism: are the open states of mammalian complex I identified by cryoEM resting states or catalytic intermediates?

**DOI:** 10.1016/j.sbi.2022.102447

**Published:** 2022-09-07

**Authors:** Injae Chung, Daniel N. Grba, John J. Wright, Judy Hirst

**Affiliations:** MRC Mitochondrial Biology Unit, University of Cambridge, The Keith Peters Building, Cambridge Biomedical Campus, Hills Road, Cambridge, CB2 0XY, UK

## Abstract

Respiratory complex I (NADH:ubiquinone oxidoreductase) is a multi-subunit, energy-transducing mitochondrial enzyme that is essential for oxidative phosphorylation and regulating NAD^+^/NADH pools. Despite recent advances in structural knowledge and a long history of biochemical analyses, the mechanism of redox-coupled proton translocation by complex I remains unknown. Due to its ability to separate molecules in a mixed population into distinct classes, single-particle electron cryomicroscopy has enabled identification and characterisation of different complex I conformations. However, deciding on their catalytic and/or regulatory properties to underpin mechanistic hypotheses, especially without detailed biochemical characterisation of the structural samples, has proven challenging. In this review we explore different mechanistic interpretations of the closed and open states identified in cryoEM analyses of mammalian complex I.

## Introduction to complex I

Respiratory complex I is a key metabolic enzyme in mammalian mitochondria [1,2]. Due to its defining roles in NADH homeostasis, respiration and oxidative phosphorylation, defects in complex I are associated with a wide range of clinical mitochondrial diseases [3–5]. Complex I catalyses oxidation of NADH and reduction of ubiquinone-10, coupled to transfer of four protons across the inner-mitochondrial membrane to generate the proton-motive force (Δp) that powers ATP synthesis and transport processes. Furthermore, mammalian complex I is a thermodynamically reversible catalyst, switching cleanly into reverse (Δp-driven ubiquinol-10:NAD^+^ oxidoreduction or ‘reverse electron transfer’) when Δp is high enough [6,7].

**Figure 1:**
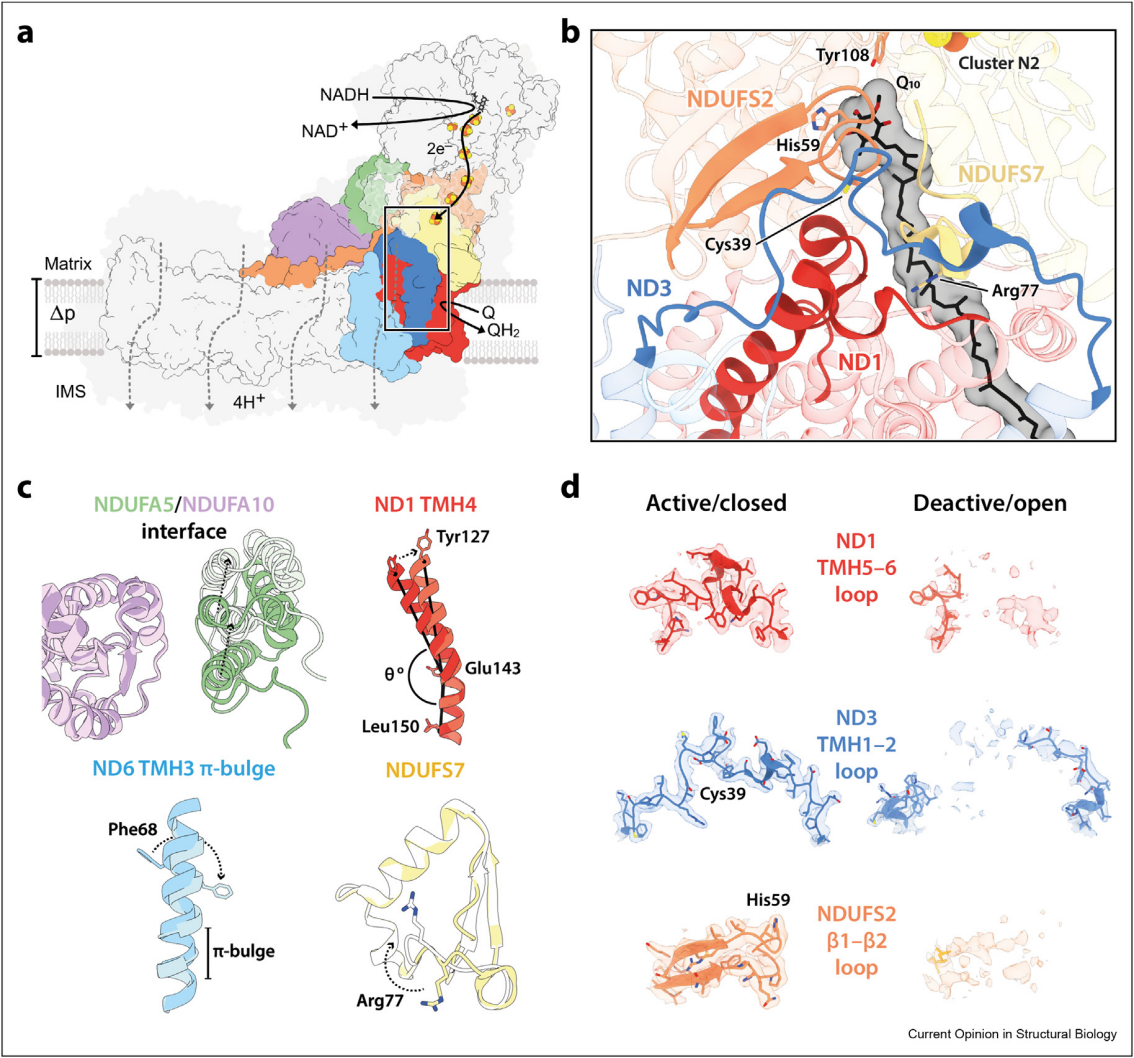
Architecture of complex I and key elements of the active/closed–deactive/open transition. **a)** A schematic overview of mammalian complex I. Key subunits involved in the active/closed–deactive/open transition are shown in colour, with the remaining core and supernumerary subunits outlined in black or shaded in grey, respectively. The ubiquinone-binding channel is indicated with a box and four proton transfer routes are shown schematically with dotted lines. **b)** A cartoon representation of the ubiquinone-binding channel in the active/closed state [PDB: 7QSL (protein), 7QSK (Q_10_)], outlining ND1-TMH4 and the ND1-TMH5–6, ND3-TMH1–2, NDUFS2-β1–β2, and NDUFS7-Arg77 loops. **c–d)** Key elements of the active/closed–deactive/open transition. Models for the deactive/open state [PDB: 7QSN] are shown in lighter shades. CryoEM densities [EMD-14133 (active/closed); EMD-14140 (deactive/open)] in **d** are shown at a map threshold of 5 in UCSF ChimeraX. Relevant residues are annotated, and subunits coloured throughout as follows: ND1, red; ND3, blue; ND6, light blue; NDUFS2, orange; NDUFS7, yellow; NDUFA5, green; NDUFA10, purple. Abbreviations: Q/QH_2_, ubiquinone/ubiquinol; Q_10_, ubiquinone-10; Δp, proton-motive force; IMS, intermembrane space; TMH, transmembrane helix.

Recent developments in single-particle electron cryomicroscopy (cryoEM) have led to an explosion of complex I structures from mammals, plants, single-cell eukaryotes and bacteria [8–22], providing unprecedented opportunities for understanding its mechanisms of catalysis and regulation. They have revealed the conserved architecture of complex I and key elements of its catalytic machinery [Figure 1a]. *Redox catalysis* occurs by fast and reversible NADH oxidation by a flavin mononucleotide at the top of the hydrophilic domain; electron transfer by a chain of ironesulphur clusters; and reduction of ubiquinone-10 in a long, amphipathic channel at the interface of the hydrophilic and membrane domains. *Proton transfer* occurs against Δp in a series of four modules; it is most likely powered by energy transfer along a chain of charged residues from the ubiquinone-binding site. The *coupling mechanism*, the least understood component, is intimately linked to ubiquinone reduction, but there is no consensus on the molecular coupling mechanisms that use redox catalysis to trigger and drive proton transfer and conserve the energy released. Notably, two recent publications [11,12] have both been entitled “the coupling mechanism of mammalian [respiratory/mitochondrial] complex I” d but the two mechanisms proposed have little in common and neither has been substantiated by coherent biochemical and biophysical analyses. Nevertheless, contrasting them highlights a key issue at the heart of the debate: are the conformational states of mammalian complex I identified by cryoEM (in both these and earlier studies) off-cycle resting states or on-cycle catalytic intermediates?

## Observation of multiple states in cryoEM analyses of mammalian complex I

As soon as cryoEM particle classification was employed to investigate the homogeneity of ‘as-prepared’ samples of mammalian complex I (grouping protein molecules according predominantly to their global conformations), it was obvious that more than one class is typically present. We described three major classes in our preparations of bovine (*Bos taurus*) complex I [8,23] but only one in the mouse (*Mus musculus*) enzyme [24]. The ovine (*Ovis aries*) and porcine (*Sus scrofa*) enzymes were resolved into four [11,25] and two [12] major classes, respectively.

One of the major classes, described as the ‘closed’ state as it exhibits the smallest apparent angle between the hydrophilic and membrane domains that form the complex I L-shape, is common to all the analyses. As expected for a catalytically relevant state, the loops that compose the ubiquinone-binding channel at the domain interface in the closed state are well ordered [Figure 1b]. Common to work on the bovine and porcine enzymes is also a well-defined ‘open’ state characterised by disorder in the ND3-transmembrane helix (TMH) 1–2, ND1-TMH5–6 and NDUFS2-β1–β2 loops that form the ubiquinone-binding channel, changes to the NDUFS7-Arg77 loop, as well as associated changes in the membrane domain involving the ND6-TMH3 and ND1-TMH4 helices [Figure 1c and d]. As a result of the disordered ubiquinone-binding channel in the open state, the domain interface has relaxed and the apparent hinge angle has opened, in a movement most clearly visualised by the relative positions of subunits NDUFA5 and NDUFA10 on the hydrophilic and hydrophobic domains, respectively [Figure 1c].

Due to variations between species, preparations, and classification strategies, different numbers of open states have been observed. An additional open class identified for the bovine enzyme has been named the ‘slack’ state because disorder in specific elements of the membrane domain opens the ND2eND4 interface and further relaxes the global structure [8,23]. Similar characteristics were observed in the major class from a cryoEM analysis of complex I from macaque (*Macaca mulatta*), which displayed only very low activity [26]. The functional competence of the slack state is thus uncertain, and we do not consider it further here. For simplicity, we focus only on the two (closed and open) states described above. Finally, we note that multiple open classes of ovine complex I have been described [11,25]. They have much in common with the open states described for the bovine and porcine enzymes, but have not been distinguished structurally in the same way. Instead, they have been described as a distribution that can be further divided by more detailed classification [25], consistent with a progressive relaxation of structural restraints (that extends to include slack-like states). The status of the distinguishing elements in ‘as-prepared’ samples of the four mammalian species discussed are summarised in Table 1. Next, we consider how the closed and open states of mammalian complex I observed in cryoEM may be interpreted and reconciled with what is known about its activity and behaviour.

### Interpretation 1: opening and closing in the active/deactive transition

**Scheme 1:**
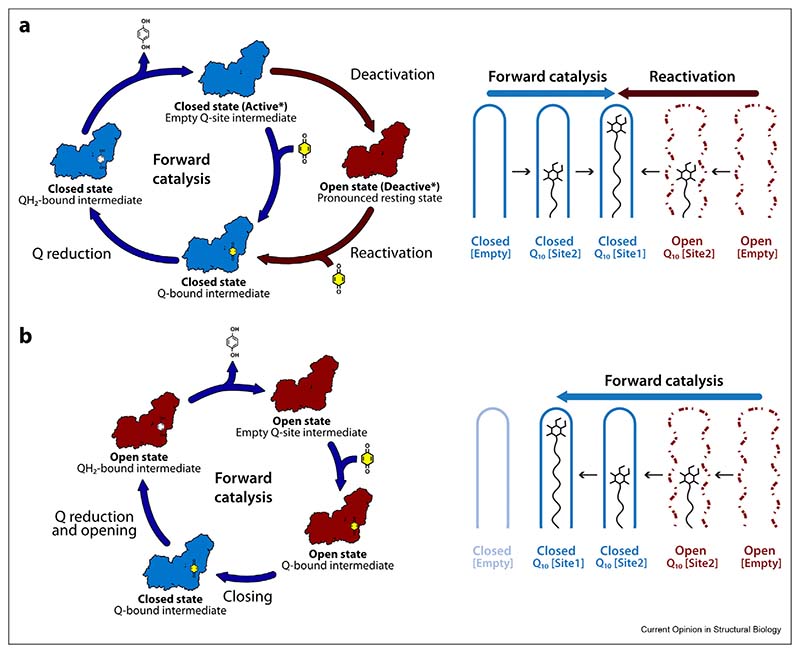
Two interpretations for the presence of open and closed states in resting preparations of mammalian complex I. Closed states are shown in blue and open states in red and ubiquinone and ubiquinol are shown in yellow and white, respectively. All the catalytic intermediates shown are expected to be predominantly reduced because the kinetics of NADH oxidation by mammalian complex I are much faster than the kinetics of ubiquinone reduction [50]. **a) *Interpretation 1***. Left, catalysis proceeds by a set of closed intermediates. When catalysis stops, the enzyme comes to rest in a closed ‘active’ resting state, which may correspond to a substrate-free intermediate. The closed/active resting state gradually deactivates to form the deactive/open resting state, an off-cycle state with a partially unstructured ubiquinone-binding channel. The active and deactive resting state structures defined by cryoEM (*) are the oxidised forms of these two resting states. Addition of substrates to the deactive enzyme stimulates closing and returns the enzyme slowly to catalysis. Right, ubiquinone-10 (Q_10_) binding in the closed channel during catalysis generates the reactive closed state with the substrate fully inserted (blue arrow); the same state is generated as ubiquinone enters the unstructured ubiquinone-binding channel in the deactive state and templates the restructuring of the site (red arrow). [Empty], [Site 2] and [Site 1] indicate the unoccupied Q_10_-binding channel, Q_10_ bound at the channel entrance, and Q_10_ bound at the reactive site in the channel, respectively. **b) *Interpretation 2***. Left, catalysis proceeds by a series of open and closed states, and when catalysis stops the enzyme rests in these states. The series of catalytic intermediates shown, based on proposals by Kampjut and Sazanov [11,41], shows how ubiquinone-binding to the open state causes the enzyme to close for ubiquinone reduction. The enzyme then opens again as the ubiquinol is released so that the substrate-free closed enzyme does not exist on the cycle. Right, during turnover ubiquinone binds to the unstructured binding site in the open state and the enzyme closes for ubiquinone reduction (blue arrow). Note that an off-cycle, deactive open state was also described by Kampjut and Sazanov [11,41] but only observed following *in vitro* treatment of the purified enzyme.

The active/deactive transition of complex I was first described by Vinogradov and co-workers [27], and later proposed to be prominent in ischaemiaereperfusion injury [28–30]. When mammalian complex I stops catalysing, it adopts the so-called ‘active’ resting state, a ‘ready-to-catalyse’ resting state. The active resting state gradually converts to the ‘deactive’ resting state, a pronounced resting state that requires reduction by NADH and reactivation by ubiquinone to return to catalysis [Scheme 1a] [27,29,31]. The two resting states can be differentiated biochemically by their sensitivity to N-ethylmaleimide (NEM), which prevents reactivation of the deactive state by derivatising ND3-Cys39 on the ND3-TMH1–2 loop [Figure 1d] [32–34]. On this basis, preparations of mammalian complex I typically comprise a mixture of the active and deactive resting states, so we proposed [23] that the closed structure corresponds to the active resting state, and the open structure to the deactive resting state. The tightly defined closed structure and its well-ordered ubiquinone-binding channel suggests it is ready to begin catalysing immediately, whereas the disordered elements of the open state are consistent with its need for restructuring and reactivation. Furthermore, ND3-Cys39 is occluded in the closed structure but in the open structure the ND3-TMH1–2 loop is disordered, consistent with exposure of Cys39 [Figure 1d]. Our initial assignment was substantiated by cryoEM analysis of a sample of bovine complex I prepared from purposefully deactivated membranes, which displayed deactive biochemical characteristics (NEM sensitivity and a catalytic lag phase during reactivation [27,33]) and which was highly active following reactivation [35]. CryoEM revealed the open structure described above as the dominant state in the deactivated sample, and a matching structure was determined similarly for purposefully deactivated mouse complex I [24].

We propose that the structural elements that change during deactivation [Figure 1 and Table 1] are unstable in the active resting state, perhaps because they are conformationally mobile during catalysis and/or because they are destabilised when the resting binding channel is occupied by water molecules or fatty acids [36] instead of ubiquinone. Therefore, they slowly relax in the resting enzyme, in a coordinated transition to the open/deactive resting state. Their closed/active conformations are recovered when substrates stimulate and template their restructuring [Scheme 1a]. Therefore, the open/deactive resting state does not feature on the catalytic cycle and sustained catalysis, including ubiquinone binding and ubiquinol release, occurs within a set of closed intermediates. Several mechanistic proposals are consistent with this interpretation [12,15,37–40], which provides a broad structural rationale for long-standing biochemical observations on the deactive state, including its sensitivity to NEM, catalytic lag phase during reactivation, and slow but spontaneous formation in ischaemic tissue that is protective against ischaemia–reperfusion injury [10,28–30].

### Interpretation 2: opening and closing as an intrinsic feature of catalysis

Kampjut and Sazanov recently proposed an alternative interpretation of the mixture of open and closed states that they observe in their ‘as-prepared’ resting ovine complex I [11,41]. Based on the fact that it is catalytically competent, they proposed that all the states observed by cryoEM in the preparation (open and closed alike) are catalytic intermediates. Consequently, they proposed that opening and closing is an intrinsic and essential part of the catalytic cycle, in which ubiquinone-binding is initiated in the open state, the enzyme closes for ubiquinone reduction, and then reopens again as ubiquinol is released [Scheme 1b]. The closed enzyme is predicted not to exist without ubiquinone/ubiquinol occupying its binding channel. Notably, ubiquinone binding to the open state to initiate closing is common to both interpretations, but it occurs more slowly (during reactivation in the presence of NADH) in *Interpretation 1* than (on the catalytic time-scale) in *Interpretation 2* [compare Schemes 1a and b]. Similarly, opening either occurs slowly during deactivation — or rapidly, during every turnover cycle.

In *Interpretation 1*, a mixture of active and deactive (and inactive) enzymes will immediately begin to turnover upon substrate addition due to the ready-to-catalyse active molecules, then increase its rate upon reactivation of the deactive molecules (inactive molecules will remain inactive). Therefore, the active/deactive model can explain the catalytic competence of a mixed population. To capture known biochemical active/deactive behaviour for ovine complex I in their proposal, Kampjut and Sazanov characterised the structure by cryoEM after incubating the detergent-solubilised enzyme at 32° C to deactivate it [11]. Four open states, proposed to be distinct from open states present in the starting preparation by destructuring of the ND6-TMH4–5 β-hairpin and the relocation of TMH4, were resolved. No closed states were observed. However, similar changes have not been observed in other deactivated mammalian enzymes [10,24,35], and these changes to ND6, together with loss of density for nearby subunit NDUFA11, may instead reflect the known instability of ovine complex I in the absence of complex III [25], exacerbated by the incubation in detergent. It is possible that ovine ND6 recovers its native structure, together with the structural elements highlighted in Figure 1, during global reactivation of the deactive enzyme. Alternatively, its altered structure may not affect catalysis, as suggested by the different positions/disorder of ND6-TMH4 in structures of all the non-mammalian species listed in Table 1.

## The challenges of combining catalysis and cryoEM

Experiments to freeze complex I onto cryoEM grids while it is catalysing have been carried out in a quest to observe the structures of the intermediates present directly — perhaps to catch the elements discussed above in different conformations. For complex I, the experiment is technically demanding due to challenges in providing sufficient electron acceptor (ubiquinone or O_2_) to sustain catalysis in a high concentration sample undergoing rapid turnover for long enough for grid preparation and freezing.

i) *Yarrowia lipolytica* complex I (2 mM) was frozen after 20–30 s at 18° C in 2 mM NADH, 200 mM decylubiquinone (a short-chain ubiquinone) and 1 mM ubiquinol oxidase [15,42]. In the single state resolved, a small decrease in the apparent inter-domain angle made the enzyme more closed relative to the single state resolved for the as-prepared, resting enzyme [15,16]. The decrease may be due to enzyme reduction or the activation of relaxed elements: the ND1-TMH4 and ND1-TMH5–6 loop conformations became closer to those in the mammalian active/closed state, while ND6-TMH3 retained its π-bulge. Importantly, no mixture of closed and open intermediates was observed.
ii) Ovine complex I (3 μM) was frozen after 20 s at 4° C in 1 mM NADH and 1 mM decylubiquinone [11]. The preparation began as a mixture of closed and open states, and no substantial shift in their ratio was caused by the substrates. It is possible that both the ‘as-prepared’ and turnover conditions produce similar mixtures. Alternatively, while closed/active molecules catalyse upon substrate addition, inactive open molecules (such as those observed in our macaque preparation) cannot. We note the specific activity for the purified ovine enzyme (5–6 units mg^−1^ [43][D. Kampjut, PhD thesis, Institute of Science and Technology Austria, 2020]) is considerably less than the value reported for complex I in ovine membranes (12–14 units mg^−1^)[43]) and also that open/deactive molecules may be slow to reactivate at 4° C.

In the future, using cryoEM to investigate the distribution of conformational states generated during catalysis by an enzyme that begins in a structurally homogenous closed/active state may provide a more definitive answer to whether open states are present on the catalytic cycle or not.

At present, we favour *Interpretation 1*: that the closed and open states observed in preparations of mammalian complex I represent the active and deactive resting states, and there is currently insufficient evidence to assign open states to the catalytic cycle. Catalytically relevant movements are likely to be tightly controlled and between well-defined and ordered conformations, rather than global relaxation processes that result in open states. We propose that the structural elements that relax during deactivation [Figure 1 and Table 1] are conformationally mobile during catalysis, and note that movement of the ND3-TMH1–2 loop has been suggested on the basis that using cross linking to restrict it was observed to decouple catalysis [44]. In the future, using the same method to restrict opening and closing movements may provide an alternate approach to evaluate their catalytic relevance.

## Insights from cryoEM analyses of non-mammalian complexes I

The fourteen ‘core’ subunits of complex I are conserved in all species and considered sufficient for catalysis. Therefore, we expect the mechanism to be conserved, and all species to catalyse via a matching set of catalytic intermediates and transition states. In contrast, different enzymes, with different thermal stabilities, supernumerary subunits and physiological environments, and isolated using different procedures, may relax differently in the absence of substrates and rest in different conformations. Therefore, we surveyed structures of complex I from plants, single-celled eukaryotes, bacteria and archaea to evaluate the status of the key structural elements that change in the mammalian complex during deactivation/opening [see Table 1].

It is clear that the simple binary nature of the active/closed and deactive/open resting mammalian enzymes does not extend to the other organisms, with many exhibiting mixed characteristics. Only one, from the ciliate *Tetrahymena thermophila*, is observed by cryoEM in a homogeneous active/closed state under resting conditions. This structure argues against opening and closing during catalysis as it contains species-specific supernumerary subunits that lock the substrate-binding site in the active/closed conformation [17]. Common to all others is the deactive-type ς bulge in ND6-TMH3 that disrupts the proposed connection between the ubiquinone-binding site and proton-translocating modules [8,11,38]. Nearby is ND1-TMH4, in the deactive-type straighter form in most species. The active-type bent form correlates partially with ordered active/active-like conformations of the ND3-TMH1–2, ND1-TMH5–6, NDUFS2 and NDUFS7 loops that form the ubiquinone-binding channel [Fig. 1b]. These varied combinations of active- and deactive-type elements may suggest each species of enzyme relaxes differently and to a different extent, from the same initial resting state when catalysis stops. For example, *Y. lipolytica* complex I has a lower energy barrier for its (limited) active-to-deactive transition than the mammalian enzyme [45,46]. In contrast, other enzymes show no evidence of undergoing a transition: *T. thermophila* complex I has been proposed to be structurally trapped in the closed state [17], and *Paracoccus denitrificans* complex I (which contains a Cys39 equivalent) displays no sensitivity to NEM [47]. Between the mammalian enzymes, the relative proportions of open and closed states observed suggest that the mouse enzyme (predominantly closed) has the highest barrier for deactivation/opening and the ovine enzyme (mostly open) the lowest. Alternatively, the different combinations of conformations may result from different species adopting different initial resting states (at different stages around the cycle) when catalysis stops, so they might provide insights into how different mobile elements change their conformations individually during catalysis.

## The ND6-P25L mouse model: rapid deactivation and unidirectional catalysis

**Scheme 2:**
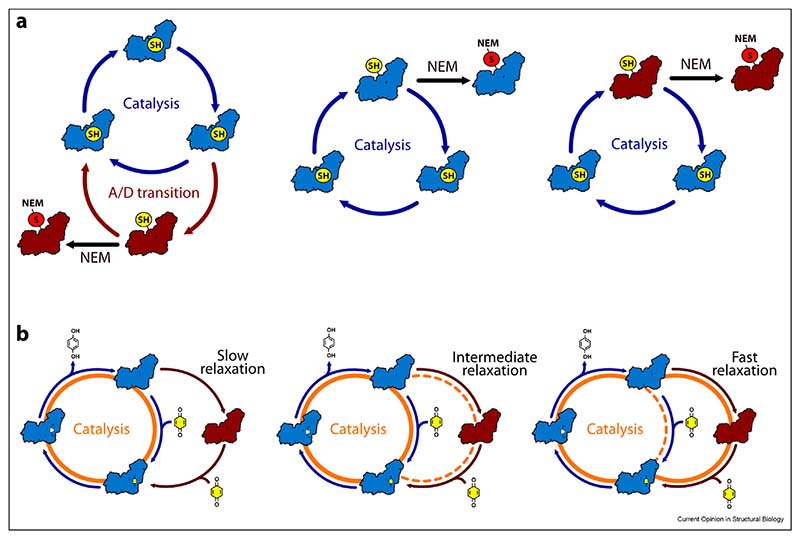
Developing a unified explanation for the behaviour of slow-relaxing and fast-relaxing variants of complex I. Closed states are shown in blue and open states in red. **a)** Simple conceptual possibilities for the status of the ND3-TMH1–2 loop and Cys39. Cys39 (SH, yellow) can be occluded, as in the closed/active resting enzyme, or exposed, as in the open/deactive resting enzyme. When exposed it is available for derivatisation by NEM (S-NEM, red). Left: Cys39 is only exposed and derivatised following relaxation into an off-cycle open/deactive state. Middle: Closed states transiently expose Cys39 during turnover allowing it to be derivatised. Right: Open states formed during catalysis expose Cys39 for derivatisation by NEM. **b)** A slow-relaxing enzyme (left, for example wild-type mouse complex I) catalyses using only closed states; the resting active/closed enzyme relaxes slowly into the deactive/open state, which reactivates slowly upon addition of substrates. In contrast, a fast-relaxing enzyme (right, for example the ND6-P25L mouse variant) most often follows the detour cycle, through an open, deactive-like state, in which enzyme opening and closing both occur on the catalytic timescale. Intermediate cases (the porcine, bovine and ovine enzymes) may follow either pathway, depending on their relative rates of relaxation and onward catalysis (middle). Catalytic pathways are highlighted in orange, with dashed lines indicating less favoured routes. ubiquinone and ubiquinol are shown in yellow and white, respectively.

The ND6-P25L variant of mouse complex I [10,48], the only mammalian complex I that has been demonstrated not to catalyse reverse electron transfer, presents both a challenge to mechanistic models and a powerful new tool to investigate the physiological roles of reverse catalysis, such as in ischaemia–reperfusion injury. CryoEM analyses revealed, surprisingly, ND6-P25L complex I in predominantly the deactive/open state [Table 1], a marked difference from the predominantly active/closed wild-type mouse enzyme. A structural rationale describing how the mutation facilitates formation of the ND6-TMH3 π-bulge and promotes deactivation was developed [10]. Functionally, the fact that (according to its NEM sensitivity) the ND6-P25L complex cannot be maintained in the active resting state but rapidly deactivates [10] explains its inability to catalyse in reverse, because even wild-type mammalian complex I is unable to begin reverse catalysis from the deactive state [7,27]. In contrast, ND6-P25L complex I catalyses in the forward (NADH:ubiquinone oxidoreduction) direction with a rate similar to that of the wild-type, although it is promptly inhibited by NEM during turnover whereas the wild-type enzyme is not. Scheme 2a illustrates some possibilities for the status of the ND3-TMH1–2 loop and Cys39 in the catalysing and resting enzymes. First [Scheme 2a, left], ND6-P25L complex I may be derivatised by NEM during turnover because it tends to drop off-cycle into deactive-like states. Intriguingly, this would not compromise forward catalysis because, in this variant, returning to the cycle is as quick and easy as departing from it. Second [Scheme 2a, middle], a closed state with Cys39 available for derivatisation may form during catalysis; it may be formed only transiently in the wild-type enzyme (protecting against derivatisation) but be stabilised in the ND6-P25L variant (promoting derivatisation). We note that the Cys39-derivatised closed/active enzyme has been proposed recently to remain catalytically active [49] but, in this case, derivatisation of a closed intermediate would not differentiate the variants. Finally [Scheme 2a, right], if open states with Cys39 exposed dominate the catalytic cycle then inhibition by NEM during turnover is easily rationalised, but the fact that the wild-type mouse enzyme is not affected is hard to explain.

Remarkably, ND6-P25L complex I cannot be switched straight from forward catalysis into reverse [10], consistent with deactivation (or the critical step in it) occurring very rapidly, on the catalytic timescale, to prevent reversal. However, forward catalysis must then be maintained through the constant formation and dissipation of off-cycle deactive-like states: ND6-P25L complex I may catalyse by a *detour* cycle, in which it leaves the main cycle and visits a deactive-like open state. The same detour cycle would be available to all species of complex I capable of deactivating/opening, and adopted increasingly as rates of ‘deactivation’ and ‘reactivation’ increase [Scheme 2b, left-to-right]. By incorporating an essentially unidirectional (substrate-dependent) return from the deactive-like open state to the main cycle, the detour cycle creates a unidirectional phenotype, unable to catalyse in reverse. Clearly, further analyses are required to explore this intriguing proposal. We close by reflecting that catalysis in such a ‘complex’ system as complex I has no simple, single pathway to follow. Instead, it explores and adopts different routes through a complicated network of states on an energy landscape that changes in response to different conditions, bioenergetics, enzyme species and variants.

## Figures and Tables

**Table 1:** The status of structural features identified to differ between the mammalian closed/active and open/deactive states in preparations of complex I from different species.

	Species	Given name	PDB/EMDB	Resolution (A)	Reference	NDUFA5/10 interface	ND6 TMH3	ND3 TMH1-2 loop	ND1 TMH5-6 loop	ND1 TMH4	NDUFS2 β1–β2 loop	NDUFS7 Arg77 loop
Mammals	*Bos taurus*	Active-apo	7QSL/14133	2.76	Chung et al. [8]	Extensive	A	A	A	A	A	A
		Deactive-apo	7QSN/14139	2.81	Chung et al. [8]	Restricted	D	D	D	D	D	D
	*Mus musculus*	Active	6ZR2/11377	3.10	Bridges et al. [9]	Extensive	A	A	A	A	A	A
		ND6-P25L	7AK6/11811	3.82	Yin et al. [10]	Restricted	D	D	D	D	D	D
	*Ovis aries*	Native-closed	6ZKO/11256	3.80	Kampjut et al. [11]	Extensive	A	A	A	A	A	A
		Native-open1	6ZKP/11257	3.20	Kampjut et al. [11]	Restricted	D	D	D	D	D	D
	*Sus scrofa*	Active (CI^Q^)	7V2C/31640	2.90	Gu etal. [12]	Extensive	A	A	A	A	A	A
		Deactive (CI^QD^)	7V2D/31641	3.30	Gu etal. [12]	Restricted	D	D	D	D	D	D
Plants	*Brassica oleracea*	Mature	7A23/11614	3.70	Soufari et al. [13]	n/a	D	D	A-like	D-like	Alt	A-like
	*Arabidopsis thaliana*	Closed	7AR8/11876	3.53	Klusch etal. [14]	n/a	D	D	A-like	D-like	[Alt]	Mixed
		Open	7AR7/11875	3.46	Klusch etal. [14]	n/a	D	D	Alt	D-like	[Alt]	Mixed
Single-cell eukaryotes	*Yarrowia lipolytica*	n/a	7O71/12742	2.40	Parey et al. [15]	n/a	D	A-like	A-like	D	A	Int
	*Tetrahymena thermophila*	Closed/Active	7TGH/25882	2.60	Zhou et al. [17]	n/a	A	A	A	A	A	A
Bacteria	*Thermus thermophilus*	n/a	6Y11/XRC	3.11	Gutierrez et al. [18]	n/a	D	[A-like]	A-like	D-like	Alt	Mixed
	*Escherichia coli*	Conformation 1	7NYR/12653	3.90	Kolata et al. [19]	n/a	D	D	[Alt]	D	[Alt]	[Mixed]
	*Thermosynechococcus vestitus*	NDH-1L	6HUM/0281	3.34	Schuller et al. [20]	n/a	D	A-like	A	A	Alt	A
		NDH-1MS	6TJV/10513	3.20	Schuller et al. [21]	n/a	D	A-like	A	A	Alt	A

All the structures listed are ‘as-prepared’ enzymes that have not been treated with substrates, inhibitors or to activate or deactivate them. In S. *scrofa* and *T. thermophila* the enzyme is contained in a supercomplex. Each structural feature is compared to its structure defined in this laboratory in the ‘active’ (A) and ‘deactive’ (D) resting states of bovine complex I [8,23,35] and residue numbers are given for the bovine enzyme. Square brackets denote poor density. **ND6 TMH3:** A, α-helical or D, π-bulge. **ND3 TMH1-2, ND1 TMH5-6 and NDUFS2 β1–β2 loops:** A, matches the bovine-A loop conformation or A-like, with only minor differences; Alt, in an alternate but ordered conformation; D, disordered. **ND1 TMH4:** A, bent helix with Y127-Cα in the bovine-A position or A-like, bent helix with Y127-Cα towards the bovine-D position; D-like, straight helix with Y127-Cα toward the bovine-A position or D, straight helix with Y127-Ca in the bovine-D position. **NDUFS7 Arg77 loop:** A, Arg pointing away from NDUFS2 and an adjacent loop or A-like with the loop retracted; Mixed, state with Arg pointing away from NDUFS2 and an adjacent β-strand; D, Arg pointing towards NDUFS2 and an adjacent β-strand. Note that for *A. thaliana* the open and closed states correspond to the absence and presence of a domain-bridging ferredoxin supernumerary subunit, respectively, and the closed state does not have the same ordered features as the (mammalian) ‘closed’ state. Note that *T. vestitus* was previously known as *T. elongatus.* Abbreviations: n/a, not applicable; XRC, X-ray crystallography.
